# Thoracic and Lumbar Vertebral Bone Mineral Density Changes in a Natural Occurring Dog Model of Diffuse Idiopathic Skeletal Hyperostosis

**DOI:** 10.1371/journal.pone.0124166

**Published:** 2015-04-21

**Authors:** Steven De Decker, Richard Lam, Rowena M. A. Packer, Ingrid M. V. L. Gielen, Holger A. Volk

**Affiliations:** 1 Department of Clinical Science and Services, Royal Veterinary College, University of London, Hawkshead lane, AL9 7TA, Hatfield, United Kingdom; 2 Department of Veterinary Medical Imaging and Small Animal Orthopaedics, Faculty of Veterinary Medicine, Ghent University, Salisburylaan 133, 9820 Merelbeke, Belgium; University of California Davis, UNITED STATES

## Abstract

Ankylosing spinal disorders can be associated with alterations in vertebral bone mineral density (BMD). There is however controversy about vertebral BMD in patients wuse idiopathic skeletal hyperostosis (DISH). DISH in Boxer dogs has been considered a natural occurring disease model for DISH in people. The purpose of this study was to compare vertebral BMD between Boxers with and without DISH. Fifty-nine Boxers with (n=30) or without (n=29) DISH that underwent computed tomography were included. Vertebral BMD was calculated for each thoracic and lumbar vertebra by using an earlier reported and validated protocol. For each vertebral body, a region of interest was drawn on the axial computed tomographic images at three separate locations: immediately inferior to the superior end plate, in the middle of the vertebral body, and superior to the inferior end plate. Values from the three axial slices were averaged to give a mean Hounsfield Unit value for each vertebral body. Univariate statistical analysis was performed to identify factors to be included in a multivariate model. The multivariate model including all dogs demonstrated that vertebral DISH status (Coefficient 24.63; 95% CI 16.07 to 33.19; *p* <0.001), lumbar vertebrae (Coefficient -17.25; 95% CI -23.42 to -11.09; *p* < 0.01), and to a lesser extent higher age (Coefficient -0.56; 95% CI -1.07 to -0.05; *p* = 0.03) were significant predictors for vertebral BMD. When the multivariate model was repeated using only dogs with DISH, vertebral DISH status (Coefficient 20.67; 95% CI, 10.98 to 30.37; *p* < 0.001) and lumbar anatomical region (Coefficient -38.24; 95% CI, -47.75 to -28.73; *p* < 0.001) were again predictors for vertebral BMD but age was not. The results of this study indicate that DISH can be associated with decreased vertebral BMD. Further studies are necessary to evaluate the clinical importance and pathophysiology of this finding.

## Introduction

Diffuse idiopathic skeletal hyperostosis (DISH) is a systemic disease characterised by calcification and ossification of ligaments and entheses [1]. Although a variety of anatomical structures can be involved, DISH most typically affects the anterior longitudinal ligament resulting in bony fusion of consecutive vertebral segments [1]. DISH is relative commonly diagnosed in people with varying prevalence among different geographical regions. Suggested risk factors in people include male gender, higher age, obesity, type 2 diabetes mellitus, hypertension and genetics [2,3]. Although DISH occurs in several animal species [4], it is increasingly reported in dogs [5–8]. In agreement with the situation in people, DISH in dogs is more common in older animals and males [5]. The prevalence in dogs is estimated to be 3.8%, with a much higher prevalence of 40.6% in the Boxer breed [6]. Socio-demographic risk factors, such as obesity and diabetes mellitus, are typically not present in Boxers. DISH in the Boxer breed has therefore been suggested to represent a natural occurring translational disease model to investigate the etiopathogenesis, genetics and treatment of DISH in people [4,5,9]. Ankylosing spinal disorders, such as ankylosing spondylitis and DISH, can be associated with alterations in bone mineral density (BMD) values in people. Although there is consensus about a decreased BMD in patients with ankylosing spondylitis [10–13], there is controversy about BMD in patients with DISH [14–16]. Earlier reports, using dual X-ray absorptiometry (DXA), suggested that DISH can be associated with increased BMD [17,18]. More recent reports however, suggest that DISH is not associated with increased BMD and that there might even be a trend towards lower BMD values in people with DISH [14–16]. It is currently unclear how BMD is best measured in people with DISH [14,16]. Although DXA is considered the method of choice to measure BMD, this diagnostic procedure is not without methodological limitations. Artificial overestimation of BMD by DXA has been suggested to explain the higher BMD values of people with DISH in earlier reports [14–16]. The aim of this study was therefore to compare vertebral BMD in Boxers with and without DISH by using computed tomography (CT) with Hounsfield unit (HU) quantification. It was hypothesized that DISH could be associated with decreased vertebral BMD.

## Materials and Methods

### Ethics statement

This study was carried out in accordance with the Royal Veterinary College’s Ethics and Welfare Committee guidelines. Because all performed CT studies were performed for clinically diagnostic purposes, no specific Ethical and Welfare Committee approval was necessary. For all included dogs, written owner consent was obtained giving permission for diagnostic procedures and use of the obtained information for research purposes.

### Study Sample

All included subjects were client-owned Boxer dogs presented at a tertiary referral veterinary hospital, the Royal Veterinary College (RVC), University of London, England. The digital medical database of the RVC was searched between 2007 and 2014 for Boxers that underwent CT of the neck, thorax or abdomen. Used search terms were Boxer and CT. Patients were excluded if (1) complete medical records or CT studies were not available for review, (2) review of the CT-studies demonstrated other ankylosing (including spondylosis deformans), traumatic, degenerative or neoplastic spinal disorders, or (3) dogs suffered from systemic disorders or received medication that could influence BMD. All medical records and CT studies were reviewed by one of the authors (SDD) to determine study eligibility. The included dogs were subsequently divided in two groups: dogs with and dogs without DISH (control group). DISH was defined according to the criteria proposed by Resnick and Niwayama [1]: (1) flowing calcification and ossification along the ventrolateral aspect of at least four contiguous vertebral bodies; (2) preservation of the intervertebral disc width and the absence of overt radiographic changes indicative of degenerative intervertebral disc disease; and (3) the absence of articular process ankylosis, sacroiliac joint erosion, sclerosis or intra-articular osseous fusion. Information retrieved from the medical records included age, gender, neutering status, presenting clinical signs, clinical history, reason for undergoing CT imaging and final diagnosis.

### Imaging and measurements

**Fig 1:**
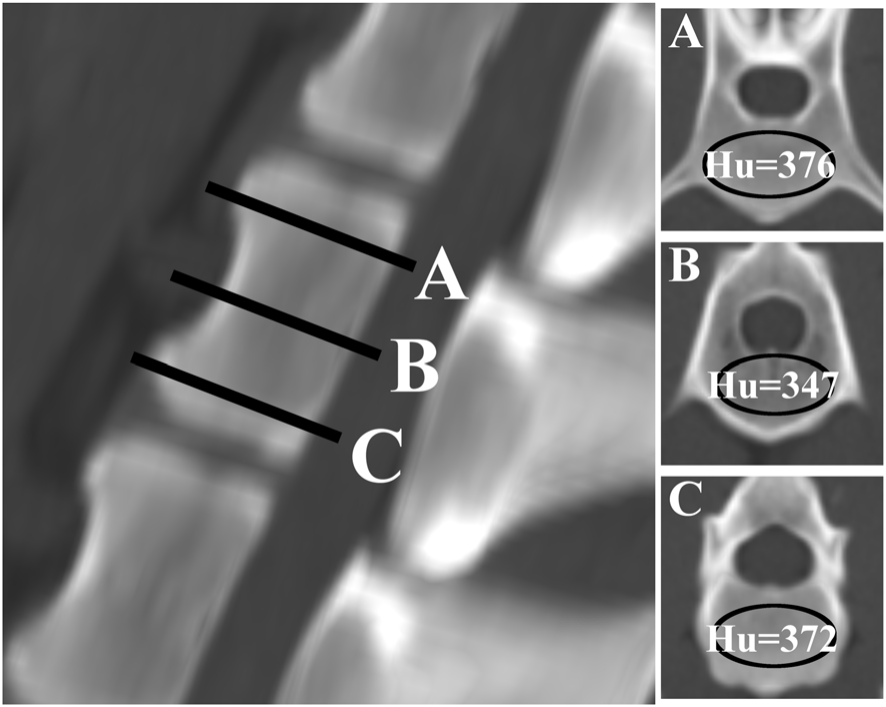
Computed tomography images demonstrating the method of determining vertebral HU as described by Schneider [19]. The left image shows the sagittal reconstructed image at the level of the first lumbar vertebra. Slices A-C represent the levels for HU measurement. Slice A was taken just inferior to the superior end plate, slice B at the middle of the vertebral body, and slice C just superior to the inferior end plate. The images at the left (A-C) represent the axial images corresponding with slices A-C. An elliptical region of interest was used to determine HU values.

All dogs underwent CT imaging under general anesthesia for a variety of clinical indications. A 16-slice helical CT scanner, equipped with automatic exposure control, was used in all cases (PQ 500, Universal Systems, Solon, Ohio or Light Speed series, GE Healthcare, Milwaukee). The CT scanners were calibrated once a week to ensure accurate CT-attenuation numbers. The CT settings for image acquisition were: helical mode, 1–2 mm slice thickness, -1 interval between slices, 140 kVp, 120 mA, 110 mm acquisition field of view, bone and soft tissue reconstruction algorithms, 512x512 matrix. After completion of the axial CT study, sagittal and coronal reconstructions were made. Standard image archiving and communication system software (Osirix Foundation, V.5.5.2 Geneva, Switzerland) was used to view imaging studies and calculate vertebral HU values. Vertebral HU was calculated for each thoracic and lumbar vertebra included in the CT study. The measurements were performed by using the protocol described and validated by Schreiber [19]. For each vertebral body, a region of interest (ROI) was drawn on the axial images at three separate locations: immediately inferior to the superior end plate, in the middle of the vertebral body, and superior to the inferior end plate (Fig 1). For each measurement, the largest possible elliptical region of interest was drawn, excluding the cortical margins, bony abnormalities, and vascular structures. The software program then automatically calculated the average HU in the ROI for each image. Values from the three axial slices were averaged to give a mean HU value for each vertebral body. To improve consistency and avoid measurement bias, all measurements were performed by a single observer (RL) unaware of the study hypothesis.

### Statistical Analysis

Unless indicated otherwise, data are presented as mean ± SEM. Univariate statistics were first performed to identify factors associated with BMD to be included in a multivariate model. T-tests were used to detect differences in BMD for categorical variables (e.g. dog with or without DISH, vertebra affected or not affected by DISH, and anatomical region (thoracic or lumbar) of assessed vertebrae, gender and neuter status of included dogs, Spearman’s rank was used to detect correlations between BMD and age and ANOVA was used to detect differences between BMD values in different vertebrae. Multivariate statistics using generalised linear mixed models for continuous outcomes were then used to identify factors associated with BMD, using the mixed models function in IBM SPSS Statistics 21. BMD was used as the response variable in all models. Each vertebra was included as the individual unit, with dog identity included as a random effect to take into account this source of non-independence. Gender, neuter status, age, dog and vertebral DISH status, and anatomical region (thoracic/lumbar) were tested. Multicollinearity was checked for in all models. Model fit was assessed using the deviance and Akaike's information criterion. A *p*-value < 0.05 was being considered statistically significant.

## Results

### Included animals

Fifty-nine Boxers with (n = 30 dogs) or without (n = 29) DISH were included in this study. These dogs were scanned for a variety of clinical indications, including diagnostic work-up of neoplastic disease (n = 46), dyspnea (n = 4), spinal pain, sepsis, suspected polytrauma after a road traffic accident (n = 2 for each), pyrexia of unknown aetiology, vasculitis and haemorrhagic diarrhea (n = 1 for each). The complete vertebral column was scanned in 18 dogs, the complete thoracic and lumbar vertebral column in 32 dogs, the complete thoracic vertebral column in 8 dogs and the complete lumbar vertebral column in 1 dog. No vertebral fractures were seen in any of the assessed CT studies. The group of affected Boxers consisted of 20 males (13 neutered) and 10 females (7 neutered) between 1.6 and 12 years old (8.3 years ± 4.9 months). The thoracic vertebral column was affected in 8 dogs, the lumbar vertebral column in 2 dogs and both the thoracic and lumbar vertebral column in 20 dogs. The group of Boxers without DISH consisted of 18 males (9 neutered) and 11 females (8 neutered) between 1.25 and 11.5 years old (6.2 years ± 6.8 months). The group of Boxers with DISH was significantly older than the group of Boxers without DISH (*p* = 0.005). There was no significant difference for gender and neutering status between both groups of dogs (*p* > 0.05).

### Vertebral bone mineral density in Boxers with and without DISH

**Fig 2:**
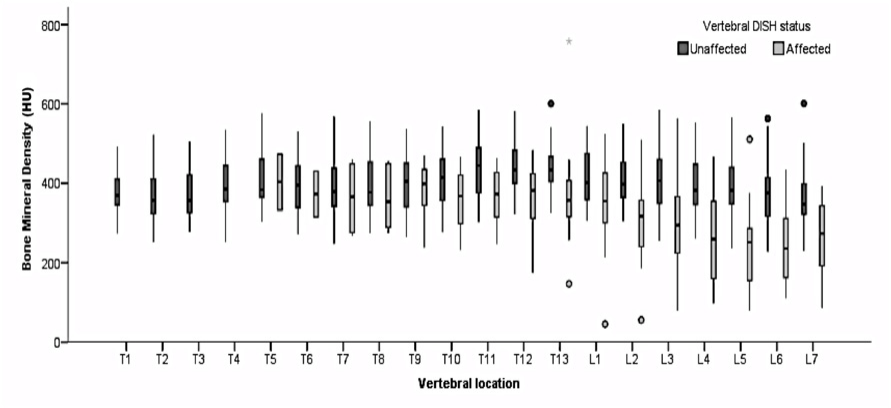
Vertebral BMD values in Boxers with (n = 30) and without (n = 29) DISH. Vertebrae affected by DISH demonstrate a significantly lower BMD compared to vertebrae not affected by DISH. This effect is most pronounced in the lumbar vertebral column. HU = Hounsfield Units.

A total of 512 vertebrae were assessed in 30 Boxers with DISH and 503 vertebrae were assessed in 29 Boxers without DISH. Of the 512 vertebrae assessed in Boxers with DISH, 232 were affected by DISH. Univariate analyses including all 59 dogs demonstrated a significantly lower vertebral BMD in dogs with DISH compared to dogs without DISH (DISH-affected: 363.45 HU ± 3.78 *vs*. DISH-unaffected: 405.43 HU ± 3.23, *p* < 0.001), BMD was significantly lower in affected vertebrae than unaffected vertebrae (affected: 336.0 HU ± 7.04 *vs*. unaffected: 397.15 HU ± 2.48, *p* < 0.001), male dogs had significantly lower BMD compared to female dogs (male: 379.88 HU ± 3.04 *vs*. female: 392.61 HU, *p* = 0.019). A significant correlation was found between age and vertebral BMD, with lower BMD in older animals (r = -0.25, *p* < 0.001). BMD was significantly lower in lumbar vertebrae than thoracic vertebrae (lumbar: 364.86 HU ± 5.47 *vs*. thoracic: 393.31 HU ± 2.72, *p* <0.001). There was no significant influence of neutering status (*p* > 0.05). When tested in the multivariate model (Table 1), three variables were still predictive of vertebral BMD: vertebral DISH status, anatomical region, and, to a lesser extent, age. Vertebrae not affected by DISH had higher BMD than vertebrae not affected by DISH, lumbar vertebrae had a lower BMD than thoracic vertebrae, and older animals had a slightly lower vertebral BMD (Fig 2).

**Table 1:** Predictors of vertebral BMD in Boxers with (n = 30) and without (n = 29) DISH.

Factor	Subcategory	Coefficient (95% CI)	SE	t	P-value
Vertebra affected by DISH?	No	24.63 (16.07–33.19)	4.36	5.64	<0.001
	Yes				
Anatomical region	Lumbar	-17.25 (-23.42 - -11.09)	3.14	-5.49	<0.001
	Thoracic				
Age (months)		-0.56 (-1.07 - -0.05)	0.26	-2.14	0.03

Univariate and multivariate statistical tests were then repeated including only the control group of 29 unaffected Boxers. At the univariable level, significant differences were found between neutered and entire dogs (neutered 415.97 HU ± 4.01 vs. entire 389.97 HU ± 5.19, *p* < 0.001) and male and female dogs (male 397.63 HU ± 4.14 vs. female HU 419.18 ± 5.01, *p* < 0.001). A significant correlation was still found between age and vertebral BMD, with BMD decreasing as age increases (r = -0.15, *p* < 0.001). No significant difference was found in BMD between lumbar and thoracic vertebrae (*p* > 0.05). When the multivariate model was repeated using only control dogs, no factors were significantly associated with BMD.

Univariate and multivariate statistical tests were then repeated including only the 30 Boxers with DISH. Vertebrae affected by DISH had significantly lower BMD compared to unaffected vertebrae (affected: 336.0 HU ± 7.04 *vs*. unaffected: 383.16 HU ± 3.69, *p* < 0.001), BMD was significantly lower in lumbar compared to thoracic vertebrae (lumbar: 330.4 HU ± 7.69 *vs*. thoracic: 380.6 HU ± 3.81, *p* < 0.001). A significant correlation was found between age and BMD, with BMD decreasing as age increases (r = -0.22, *p* < 0.001). No significant associations were found for gender or neutering status (*p* > 0.05). When the multivariate model was repeated using only dogs affected by DISH, vertebral DISH status and anatomical region were again significant predictors for vertebral BMD but age was not (Table 2).

**Table 2:** Predictors of vertebral BMD in 30 Boxers with DISH.

Factor	Subcategory	Coefficient (95% CI)	SE	t	P-value
Vertebra affected by DISH?	No	20.67 (10.98–30.37)	4.94	4.19	<0.001
	Yes				
Spinal region	Lumbar	-38.24 (-47.75 - -28.73)	4.84	-7.90	<0.001
	Thoracic				

## Discussion

This study evaluated vertebral HU as a measure for BMD in Boxers with and without a radiological diagnosis of DISH. The results of this study suggest that DISH in dogs can be associated with decreased vertebral BMD. More specifically, our findings indicate that occurrence of DISH and a lumbar localisation could be considered the most reliable and strongest variables to predict low BMD in an individual vertebra (Fig 2). Although dogs and people with DISH are usually asymptomatic, a low vertebral BMD could be of clinical importance. Several studies have documented an increased risk of vertebral fractures in people with DISH [16,20–23]. Affected people can develop severe and unstable vertebral fractures after relative minor trauma or low-energy impacts [20–23]. Although the exact reason for this increased fracture risk is currently unknown, it is postulated that increased vertebral stiffness associated with DISH causes decreased flexibility and increased biomechanical stress on the vertebral column. This is expected to interfere with normal energy distributing along the affected vertebral segment, making it more vulnerable to fracture [20,22]. The relationship between BMD, bone strength and fracture risk is well documented [19, 24–26]. Fractures associated with decreased BMD are commonly referred to as fragility fractures [13,25]. It is therefore possible that decreased BMD in patients with DISH could contribute to the increased risk of vertebral fractures. This is supported by the findings of Diederichs who found a decreased vertebral BMD in men with DISH and vertebral fractures compared to men with DISH but without fractures [16]. It is however important to note that none of the dogs in this study demonstrated clinical or radiological evidence of a vertebral fracture. It is therefore, based on the results presented here, still unclear if a lower vertebral BMD associated with DISH contributes indeed to an increased fracture risk.

Although it is currently unclear why dogs with DISH demonstrate decreased vertebral BMD, several hypotheses can be considered. DISH is a systemic disorder and it is therefore possible that decreased vertebral BMD represents an expression of altered bone metabolism in affected dogs. Ankylosing spondylitis, a disorder sharing similarities with DISH, is associated with decreased vertebral BMD and fragility fractures in people [11–13]. Several risk factors have been suggested to be associated with decreased BMD in patients with ankylosing spondylitis [13,27]. These include increased levels of inflammatory cytokines, such as IL-1, IL-6, IL-17 and TNF-α [27–29], alterations in Vitamin D metabolism [30], immobilization, and reduced physical activity [13]. In contrast to ankylosing spondylitis, DISH is considered a non-inflammatory disease and involvement of inflammatory cytokines is therefore considered unlikely to contribute to decreased BMD in the dogs presented here. It is possible that decreased vertebral BMD in the evaluated dogs with DISH is related to altered biomechanics of the vertebral column. As mentioned earlier, vertebral fusion associated with DISH causes decreased flexibility and increased stiffness of the affected vertebral segments [5,20]. This can eventually result in altered biomechanical loading of affected individual vertebral bodies. Physical exercise and mechanical stimulation are essential for increasing or maintaining bone mass and strength [31]. Loss of biomechanical function results in altered bone metabolism characterised by decreased bone formation and increased bone resorption [32,33]. This process is also referred to as disuse osteoporosis [26]. It can therefore be hypothesized that abnormal mechanical loading of DISH-affected vertebral bodies could result in alterations of bone metabolism with loss of trabecular bone mass. In the study presented here, DISH status and a lumbar localisation of individual vertebrae were the strongest independent variables to predict vertebral BMD in dogs with DISH. Both findings support the hypothesis that focal biomechanical factors could influence individual vertebral BMD values in dogs with DISH. The fact that vertebrae affected by DISH had lower BMD compared to vertebrae without DISH indicates that, although DISH is a systemic disease, trabecular bone loss associated with DISH seems to be a focal instead of a more generalised process. The thoracic vertebral column is a rather rigid structure fixed by the ribcage, while the lumbar vertebral column is considered more mobile. It is therefore possible that DISH affecting the lumbar vertebral column disrupts the spinal biomechanical properties to a greater extent than DISH affecting the thoracic vertebral column.

Vertebral BMD can be assessed by a number of techniques, such as DXA, quantitative computed tomography (QCT) or CT. Although DXA is considered the gold standard to evaluate BMD in people [34], recent studies have suggested that DXA produces unreliable and artificially increased BMD values in people with DISH [14–16]. DXA measures two-dimensional areal BMD as a sum of all attenuating tissues in the beam projection and does not take paravertebral calcifications into account [14–16]. Because the ossified anterior longitudinal ligament projects in the field of view during standard DXA scanning, an overestimated vertebral BMD value is measured in people with DISH [14]. Assessing BMD by QCT has the advantage that measurements can be limited to specific ROIs, such as the vertebral body [35]. Disadvantages of QCT are the labor-intensive character of this procedure, the requirement of an imaging phantom or angle-corrected ROI measurement of bone, muscle, and fat at multiple levels, it is a rather costly procedure and requires additional time and radiation exposure [35]. This has limited the clinical applications of QCT and it is now mainly used in a musculoskeletal research setting [36]. Assessing vertebral BMD on CT scans with HU quantification is being reported with increasing frequency in people [19, 36–41]. Due to continuous technical developments, vertebral BMD can be reliably assessed with CT imaging with good correlations between vertebral HU, BMD assessed by DXA, and osseous compressive strength [19,36,41]. Similar to the dogs included in this study, Pickhart suggested that for people undergoing a CT study for other clinical indications, an opportunity exists for vertebral BMD screening without additional imaging, costs, patient time, equipment, software, or radiation exposure [37,40]. Other advantages of measuring vertebral BMD on CT scans are the negligible amount of training required to perform the measurements, the measurements can be applied retrospectively, and are not dependent on selected tissue window or IV-contrast administration [19,36,37,40,41].

This study is limited by its retrospective nature, which complicated standardization of studied subjects. Only one specific dog breed was studied, which created the opportunity to investigate a homogeneous animal population with comparable body conformation, size, and weight. Included dogs suffered however from a variety of diseases, which limited the opportunity to assess biomarkers of bone metabolism between both groups of dogs. The group of Boxers with DISH was significantly older than the Boxers without DISH. In agreement with findings in people [42], higher age was significantly associated with lower BMD values. Although this could potentially have influenced our findings, results of multivariate statistical analysis indicated that, when considering all included dogs, DISH-status, lumbar anatomical region and higher age were separate variables each independently predictive for decreased BMD (Table 1). Furthermore, the magnitude of this effect was much higher for vertebral DISH status and lumbar anatomical region compared to a very small quantitative effect for age (Table 1). We therefore feel confident that the difference in age between both groups of dogs did not influence the conclusions of this study. Most vertebral fractures associated with DISH occur in the cervical vertebral column [20,21] and BMD evaluation of this anatomical region was not performed. Cervical vertebral BMD was not evaluated in this study because the cervical vertebral column was only included in a limited number of CT studies and DISH typically does not affect the cervical vertebral column in dogs [6].

In conclusion, the results of this study suggest that DISH can be associated with decreased vertebral BMD in dogs. Further research is necessary to evaluate if this information can be extrapolated to people, to determine the clinical relevance of this finding, and to assess the underlying pathophysiology of trabecular bone loss in patients with DISH.
